# Supplementary material for: Human cytomegalovirus deploys molecular mimicry to recruit VPS4A to sites of virus assembly
Source: PLoS Pathog. 2024 Jun 20;20(6):e1012300. doi: 10.1371/journal.ppat.1012300 (PMC11218997; doi:10.1371/journal.ppat.1012300)
Supplement: S1 Table — As quantitated by isothermal titration calorimetry (ITC). Data for independent experiments are shown. For all, the cell contained human VPS4 MIT domain (residues 1–84) and pUL71 is from HCMV unless stated otherwise.–, no binding detected. (DOCX) [file ppat.1012300.s001.docx]

**S1 Table.** **Thermodynamic properties of the interactions with VPS4 MIT domain**. As quantitated by isothermal titration calorimetry (ITC). Data for independent experiments are shown. For all, the cell contained human VPS4 MIT domain (residues 1-84) and pUL71 is from HCMV unless stated otherwise. –, no binding detected.

| **Titrant** | **[Titrant] in syringe (M)** | **[VPS4 MIT] in cell (M)** | **K_D_ (M)** | **∆H (kcal/mol)** | **∆G (kcal/mol)** | **-T∆S (kcal/mol)** | **N (sites)** |
| --- | --- | --- | --- | --- | --- | --- | --- |
| GST-pUL71 (283–361) | 8.00E-04 | 5.00E-05 | 3.07E-06 | -8.73 | -7.52 | 1.21 | 0.949 |
|  | 7.00E-04 | 6.60E-05 | 2.60E-06 | -9.36 | -7.61 | 1.75 | 1.05 |
| GST-pUL71 (P315+318A) | – | – | – | – | – | – | – |
|  | – | – | – | – | – | – | – |
| GST-CHMP6 | 6.92E-04 | 6.50E-05 | 4.76E-06 | -2.02 | -7.26 | -5.25 | 0.786 |
|  | 7.14E-04 | 6.68E-05 | 6.32E-06 | -2.72 | -7.09 | -4.37 | 0.894 |
| CHMP6 peptide | 8.00E-04 | 7.00E-05 | 5.25E-06 | -0.844 | -7.2 | -6.36 | 1 |
|  | 6.92E-04 | 6.50E-05 | 5.80E-06 | -2.04 | -7.15 | -5.11 | 0.79 |
|  | 1.00E-03 | 6.80E-05 | 1.84E-05 | -1.32 | -6.46 | -5.14 | 1.1 |
|  | 1.00E-03 | 6.85E-05 | 1.53E-05 | -1.1 | -6.57 | -5.46 | 1.17 |
| pUL71 (310–325) peptide | – | – | – | – | – | – | – |
|  | – | – | – | – | – | – | – |
|  | – | – | – | – | – | – | – |
| pUL71 (300–325) peptide | 1.00E-03 | 7.00E-05 | 5.53E-06 | -6.33 | -7.17 | -0.845 | 1.32 |
|  | 1.00E-03 | 7.00E-05 | 5.99E-06 | -5.48 | -7.13 | -1.65 | 1.46 |
|  | 1.00E-03 | 6.80E-05 | 5.17E-06 | -7.09 | -7.21 | -0.125 | 1.27 |
|  | 1.00E-03 | 6.96E-05 | 5.68E-06 | -5.98 | -7.16 | -1.18 | 1.47 |
| GST-pUL71 (283–336) | 8.00E-04 | 5.00E-05 | 2.83E-06 | -9.79 | -7.57 | 2.22 | 0.825 |
|  | 7.34E-04 | 6.89E-05 | 3.21E-06 | -10.3 | -7.5 | 2.81 | 0.995 |
| GST-pUL71 (300–361) | 8.00E-04 | 5.00E-05 | 2.94E-06 | -10.2 | -7.55 | 2.65 | 0.794 |
|  | 7.71E-04 | 6.89E-05 | 3.01E-06 | -9.97 | -7.53 | 2.44 | 0.953 |
| GST-pUL71 (310–336) | – | – | – | – | – | – | – |
|  | – | – | – | – | – | – | – |
|  | – | – | – | – | – | – | – |
| GST-pUL71 (300–325) | 1.00E-03 | 6.00E-05 | 2.11E-06 | -6.56 | -7.74 | -1.19 | 1.22 |
|  | 1.00E-03 | 6.00E-05 | 2.39E-06 | -6.76 | -7.67 | -0.909 | 1.21 |
| pUL71 (300–325) I307R peptide | – | – | – | – | – | – | – |
|  | – | – | – | – | – | – | – |
| HHV6 pUL71 peptide | 1.00E-03 | 6.85E-05 | 2.59E-05 | -6.14 | -6.26 | -0.117 | 1.16 |
|  | 1.00E-03 | 6.96E-05 | 2.96E-05 | -6.45 | -6.18 | 0.27 | 1.22 |
|  | 1.00E-03 | 1.93E-05 | 3.72E-05 | -8.91 | -6.04 | 2.87 | 1.17 |
