# Supplementary figures and images for: Human cytomegalovirus deploys molecular mimicry to recruit VPS4A to sites of virus assembly
Source: PLoS Pathog. 2024 Jun 20;20(6):e1012300. doi: 10.1371/journal.ppat.1012300 (PMC11218997; doi:10.1371/journal.ppat.1012300)

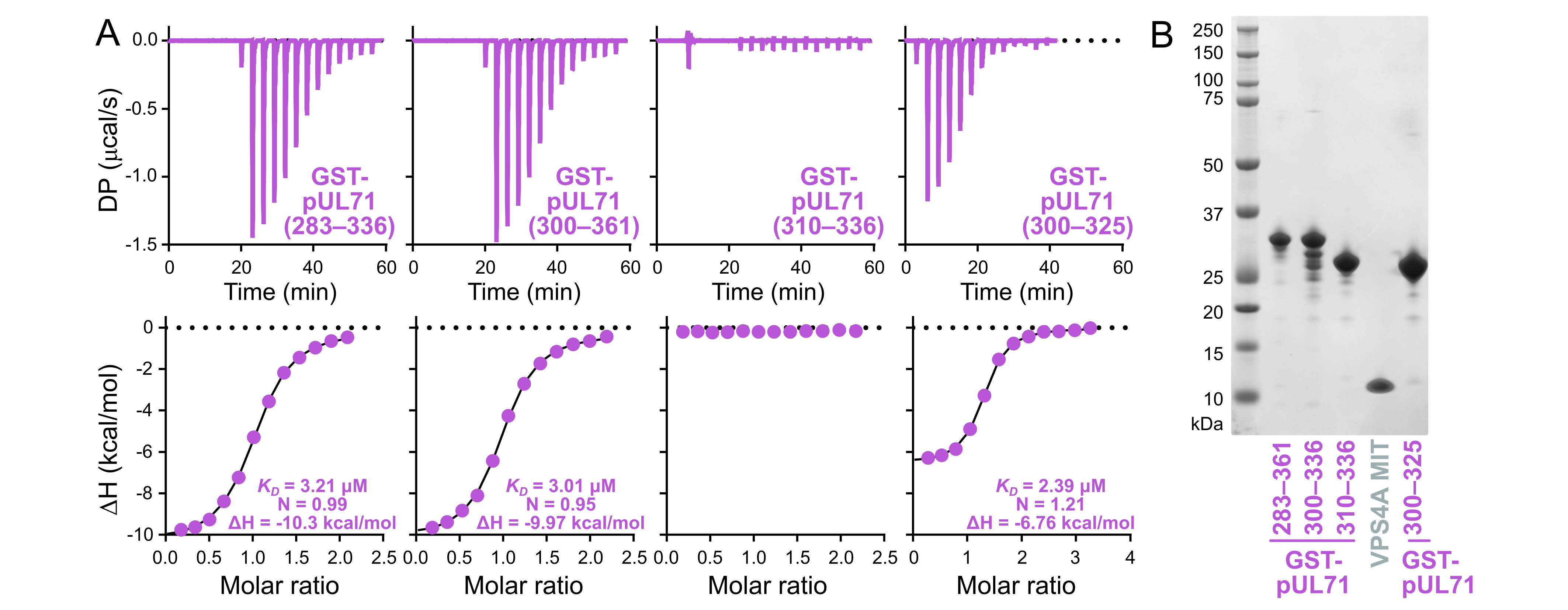

Supplement: S1 Fig — (A) ITC analysis of the interaction between purified VPS4A MIT domain and GST-tagged truncations of the pUL71 C-terminal tail. (B) Coomassie-stained SDS-PAGE of purified GST-tagged pUL71 C-terminal truncations and VPS4A MIT domain used for ITC analysis. (PNG) [file ppat.1012300.s002.png]

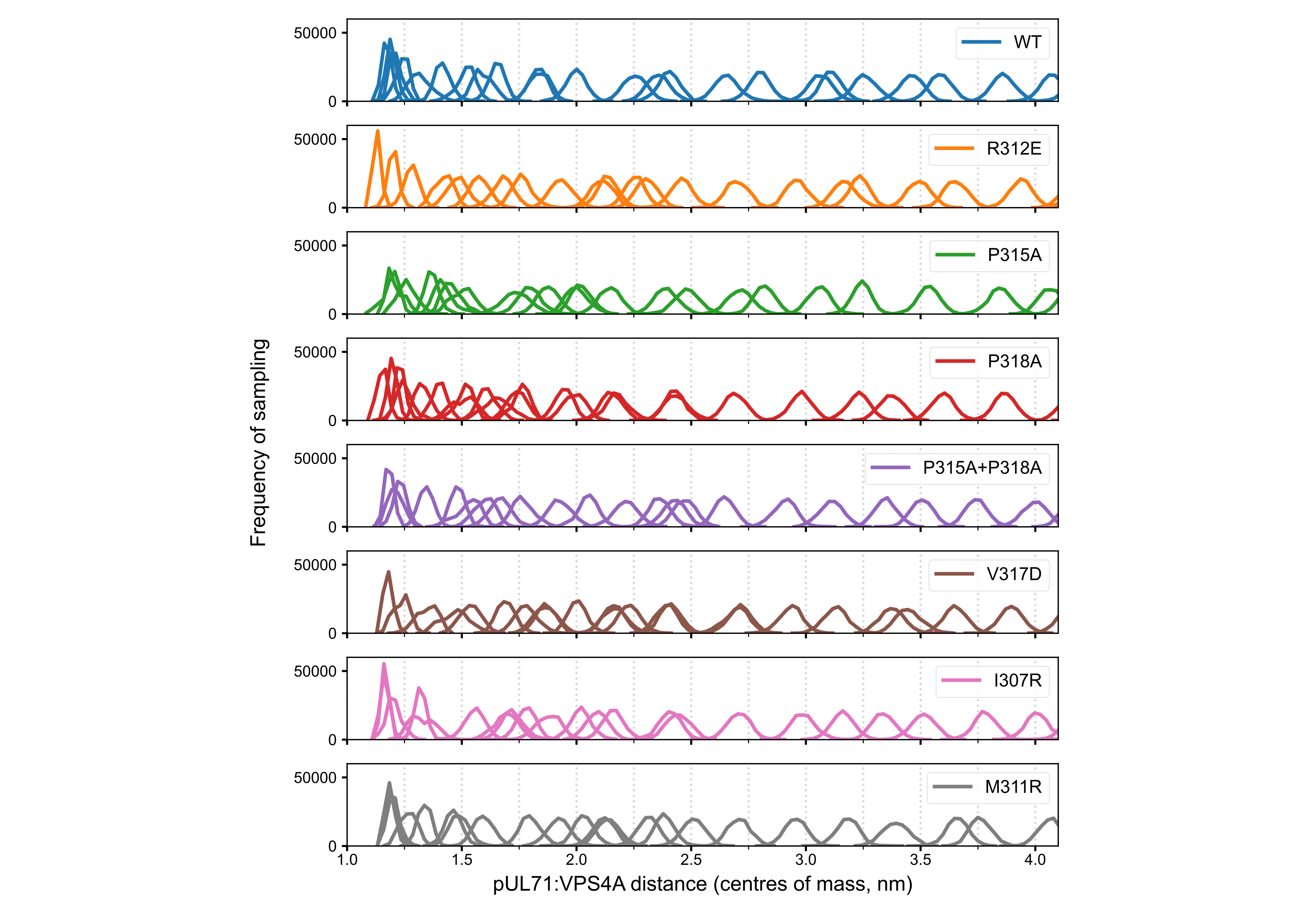

Supplement: S2 Fig — Histograms representing the distribution of pUL71:VPS4A centre-of-mass distances sampled in individual simulations along the reaction coordinate are shown for each pUL71 mutant. (PNG) [file ppat.1012300.s003.png]

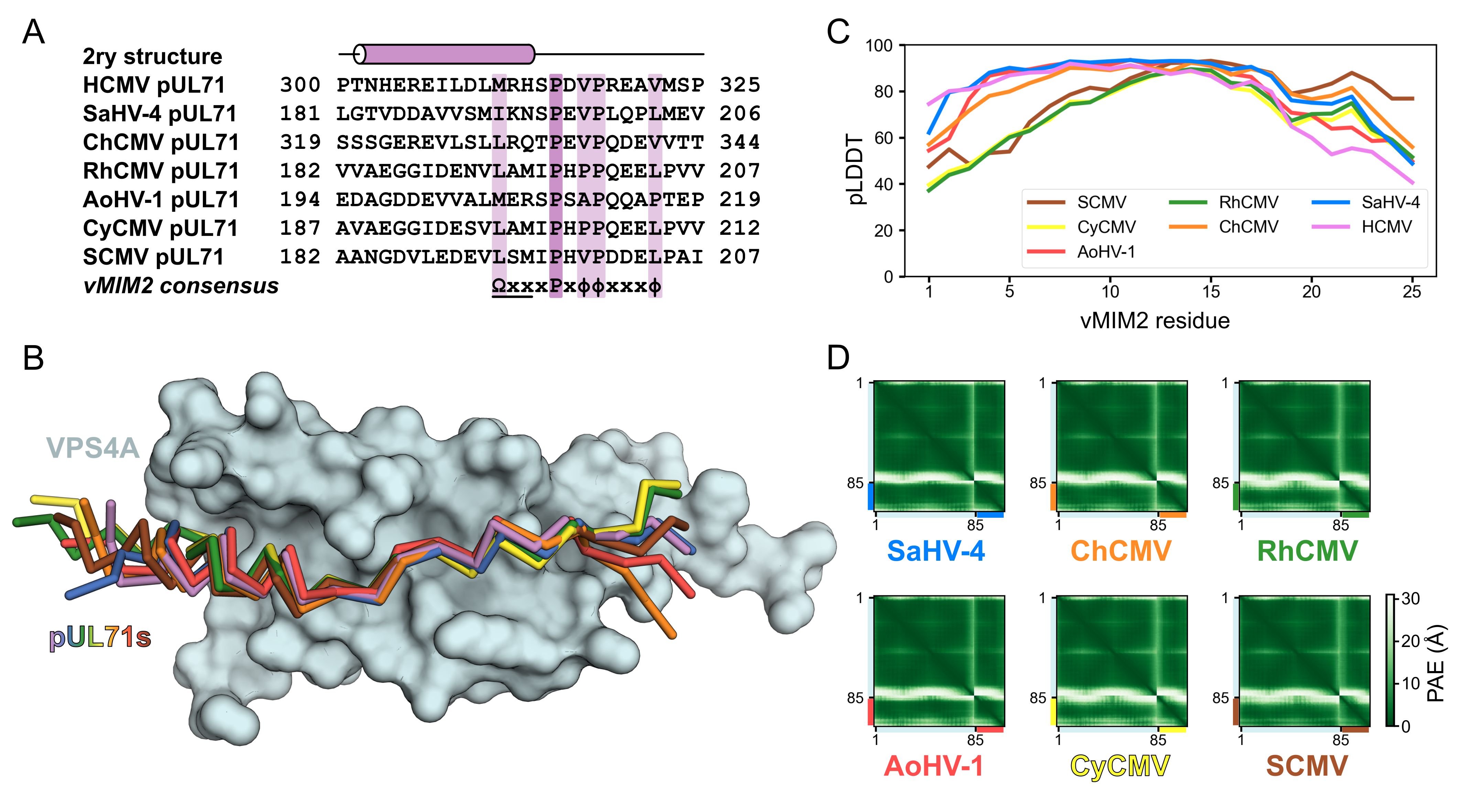

Supplement: S3 Fig — (A) Alignment of vMIM2 sequences of pUL71 homologues from primate cytomegaloviruses: saimiriine betaherpesivirus 4 (SaHV-4), chimpanzee cytomegalovirus (ChCMV), rhesus macaque cytomegalovirus (RhCMV), aotine betaherpesvirus 1 (AoHV-1), cynomolgus macaque cytomegalovirus (CyCMV), and simian cytomegalovirus (SCMV). The secondary structure of the pUL71 predicted structure is shown above. The pUL71 homologue vMIM2 consensus sequence is shown below, where Ω denotes a large hydrophobic residue, x denotes any residue, ϕ denotes a small hydrophobic residue (including proline), and where the underlined residues are within an α-helix. (B) Superposition of the predicted structure pUL71 vMIM2s (Cα traces) from SaHV-4 (blue), ChCMV (orange), RhCMV (green), AoHV-1 (red), CyCMV (yellow) and SCMV (brown) onto the prediction of human cytomegalovirus (HCMV, violet) in complex with human VPS4A MIT domain (cyan molecular surface). Predictions were performed using the VPS4A MIT domain sequence from the cognate host species for each virus, but for clarity only the human VPS4A MIT domain is shown. (C) Per-residue pLDDT scores of residues in the vMIM2s are shown. (D) PAE matrices for predicted pUL71 vMIM2:VPS4 MIT domain complexes. (PNG) [file ppat.1012300.s004.png]

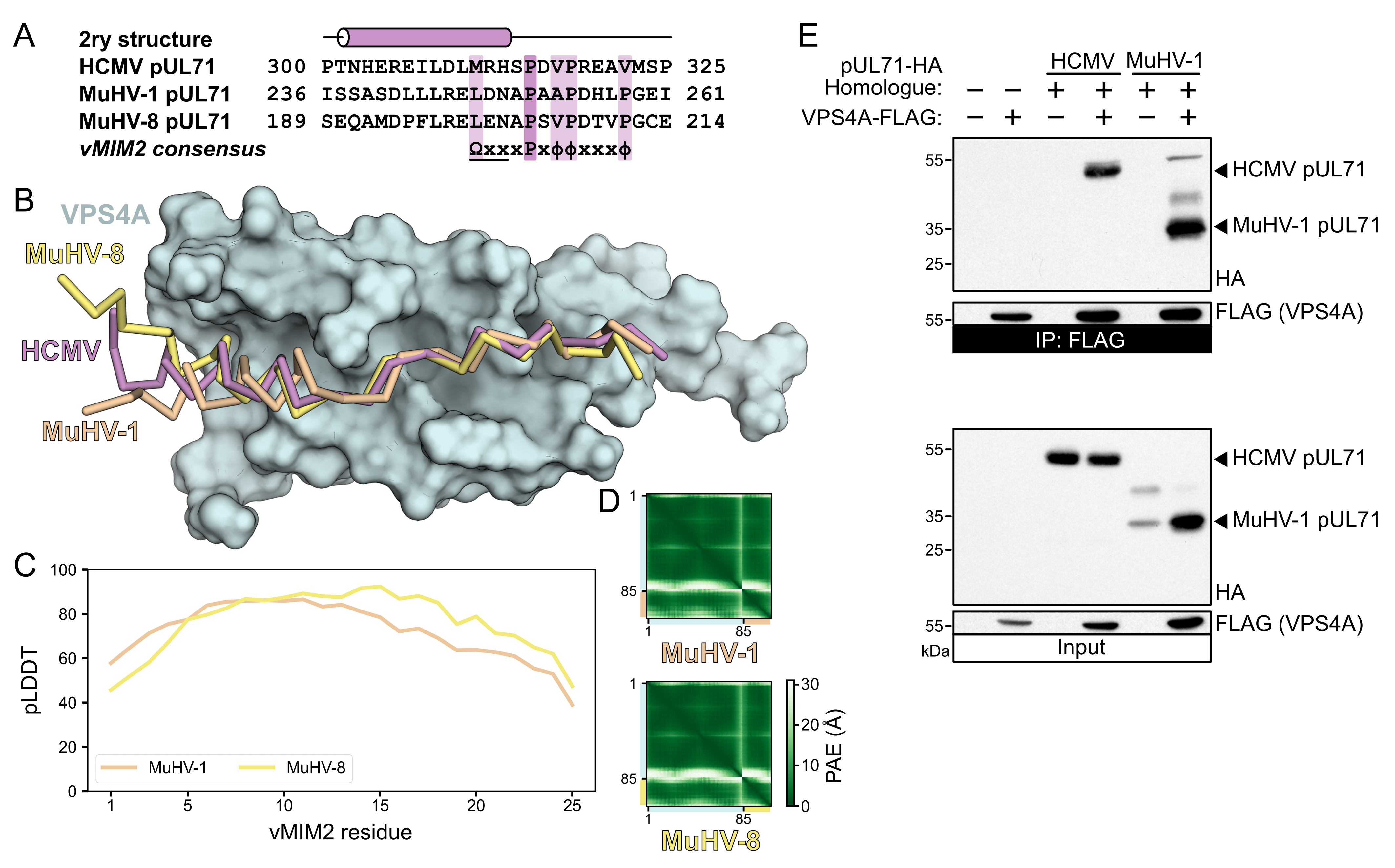

Supplement: S4 Fig — (A) Alignment of vMIM2 sequences of pUL71 homologues from mouse cytomegalovirus (MuHV-1) and the England isolate of rat cytomegalovirus (MuHV-8). The secondary structure of the pUL71 predicted structure is shown above. The pUL71 homologue vMIM2 consensus sequence is shown below, where Ω denotes a large hydrophobic residue, x denotes any residue, ϕ denotes a small hydrophobic residue (including proline), and where the underlined residues are within an α-helix. (B) Superposition of the predicted structures of pUL71 vMIM2s (Cα traces) from MuHV-1 (tan) and MuHV-8 (yellow) onto the prediction of human cytomegalovirus pUL71 (HCMV, violet) in complex with the human VPS4A MIT domain (cyan molecular surface). Predictions were performed using the VPS4A sequence from the cognate host species for each virus, but for clarity only the human VPS4A MIT domain is shown. (C) Per-residue pLDDT scores of residues in the vMIM2s are shown. (D) PAE matrices for predicted pUL71 vMIM2:VPS4 MIT domain complexes. (E) Anti-FLAG immunoprecipitation (IP) from cells co-transfected with human VPS4A-FLAG and HA-tagged pUL71 from HCMV and MuHV-1. Samples were immunoblotted using antibodies as shown. (PNG) [file ppat.1012300.s005.png]

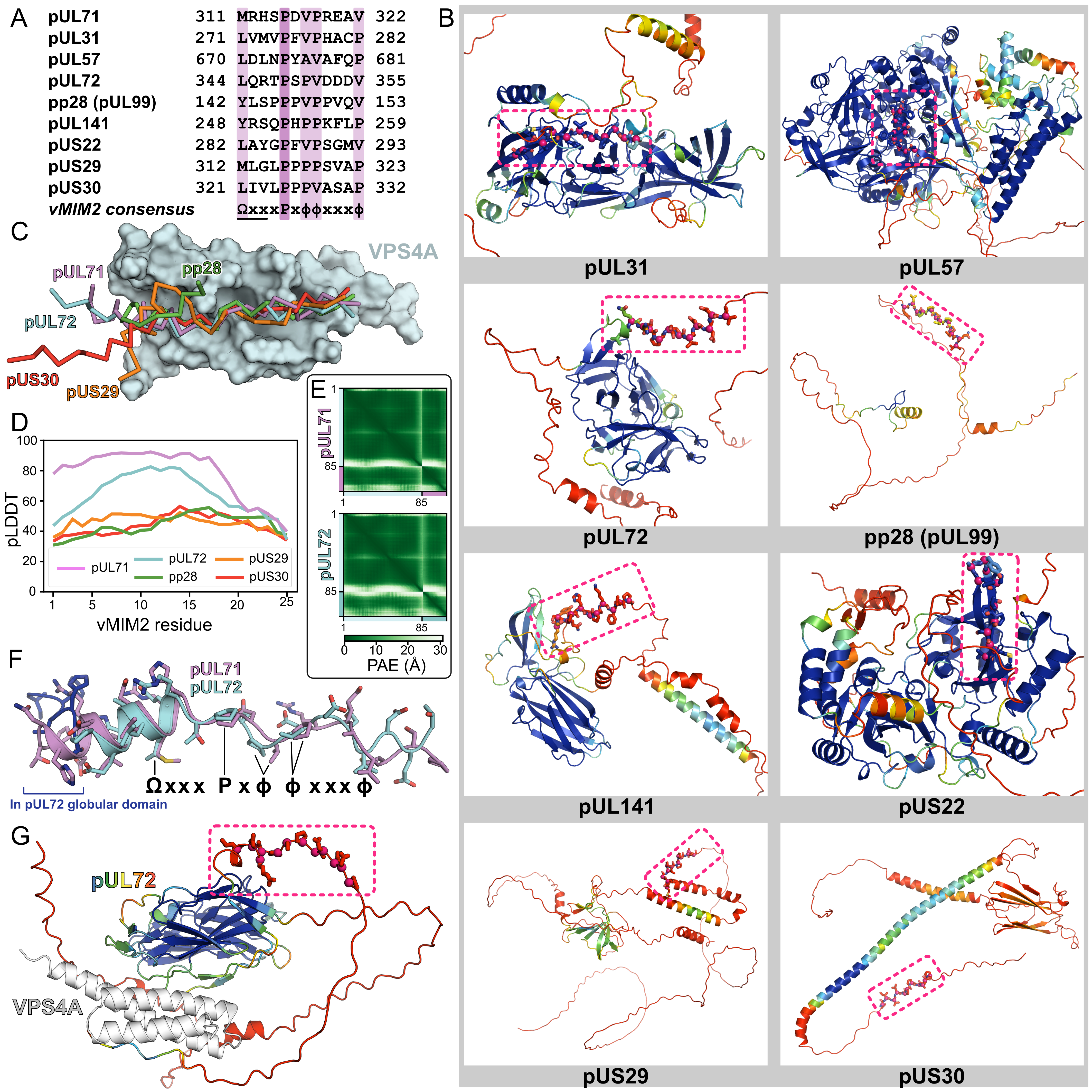

Supplement: S5 Fig — (A) Alignment of regions from HCMV proteins that match the vMIM2 consensus sequence. The vMIM2 consensus sequence is shown below, where Ω denotes a large hydrophobic residue, x denotes any residue, ϕ denotes a small hydrophobic residue (including proline), and where the underlined residues are within an α-helix. (B) AlphaFold2 predicted structures [45] of HCMV proteins containing potential vMIM2s. Predicted structures are shown as ribbons coloured by prediction confidence, from red (pLDDT = 50, low confidence) to blue (pLDDT = 90, high confidence). Regions that match the vMIM2 consensus are highlighted, with side chains shown and Cα atoms represented as pink spheres. The potential vMIM2 sequences of pUL31, pUL57 and pUS22 lie within well-ordered regions and would be inaccessible to VPS4A. The potential vMIM2 of pUL141 is in an extracellular region of the protein and would be similarly unable to engage the cytosolic MIT domain of VPS4A. (C) Superposition of the structures of potential vMIM2s (Cα traces) from pUL71 (residues 300–325, violet), pUL72 (residues 333–358, cyan), pp28 (a.k.a. pUL99; residues 131–156, green), pUL29 (residues 301–326, orange) and pUS30 (residues 321–332, red) in complex with the human VPS4A MIT domain (cyan molecular surface) as predicted using AlphaFold-Multimer version 2.3.1. For clarity only the human VPS4A MIT domain from the predicted complex with pUL71 is shown. (D) Per-residue pLDDT scores of residues in the potential vMIM2s are shown. All except pUL71 and pUL72 are predicted with very low confidence. (E) PAE matrices for pUL71 vMIM2 and potential pUL72 vMIM2 in complex with VPS4 MIT domain. (F) Comparison of predicted conformations of the vMIM2 from pUL71 (violet) and the potential vMIM2 from pUL72 (cyan) in complex with the VPS4A MIT domain (omitted for clarity). Residues that are predicted to form part of the well-ordered globular domain of pUL72 are highlighted in dark blue. The vMIM2 consensus is shown below, with key residues ide [file ppat.1012300.s006.png]

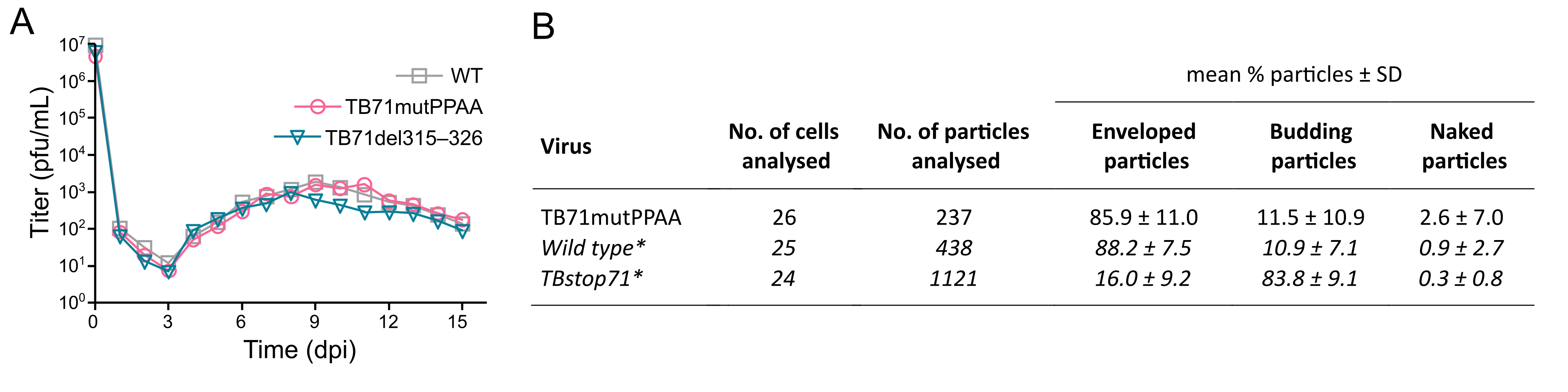

Supplement: S6 Fig — (A) Mutations in the pUL71 vMIM2 motif do not affect virus release. MDMs were infected (MOI 3) with HCMV WT (□), TB71mutPPAA (○) or TB71del315–326 (▽). The supernatant of infected cells was harvested at the indicated times post infection and the virus yield was determined by titration on HFFs. Data is shown from one experiment. Virus yields of the inocula are given at time zero. (B) Virus morphogenesis of TB-71mutPPAA in MDMs 6 days post-infection was analysed by electron microscopy and quantified from 26 cells. Relative numbers represent percent of enveloped particles, non-enveloped particles attached to membranes (budding particles), and non-enveloped particles (naked particles) at the cVACs of infected cells. *Data from equivalent experiments performed using MDMs infected with wild-type and TBstop71 HCMV are shown for comparison [29]. (PNG) [file ppat.1012300.s007.png]

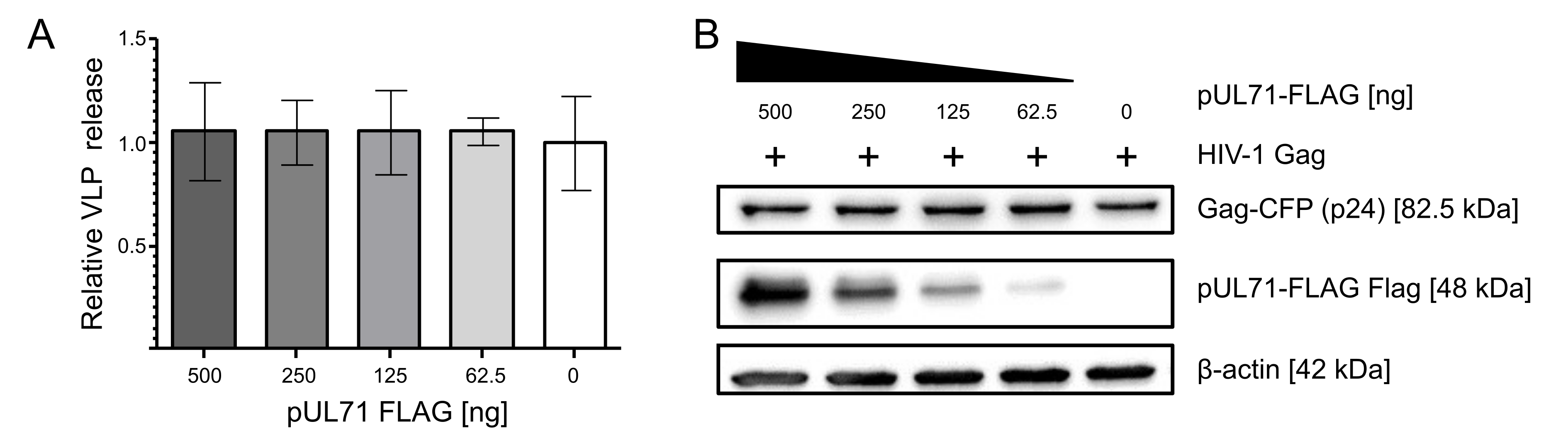

Supplement: S7 Fig — Virus-like particle (VLP) release was monitored following transfection of HEK293 cells with Gag-CFP (500 ng) and indicated amounts of pUL71-FLAG. (A) VLP release was assessed by monitoring abundance of Gag-CFP in supernatants (VLPs) relative to the corresponding whole cell lysates (WCLs) at 48 hours post transfection using a p24 (Gag capsid domain) ELISA. Data show the mean and standard deviation of the relative VLP release (p24 from VLPs / WCLs) from four individual experiments for each condition. Statistical analysis by a two tailed T-test showed no significant difference in VLP release when pUL71 is expressed. (B) Representative immunoblots from WCLs detecting expression of Gag-CFP using anti-p24 antibody, pUL71-FLAG using anti-FLAG antibody and β-actin using anti-actin antibody. (PNG) [file ppat.1012300.s008.png]
